# A comparison of three column agglutination tests for red blood cell alloantibody identification

**DOI:** 10.1186/s13104-020-04974-x

**Published:** 2020-03-04

**Authors:** Siska Blomme, Emilie De Maertelaere, Eline Verhoye

**Affiliations:** 1 Clinical Department of Laboratory Medicine, University Hospitals, Herestraat, 49 3000 Leuven, Belgium; 2 Clinical Department of Laboratory Medicine, University Hospitals, St. Pietersnieuwstraat 33, 9000 Ghent, Belgium; 3 Clinical Departments of Laboratory Medicine, AZ Delta Hospital, Ardooisesteenweg 276, 8800 Roeselare, Belgium

**Keywords:** Column agglutination technique, Red blood cell antibodies, Alloantibody identification, Transfusion medicine, Pre-transfusion testing

## Abstract

**Objective:**

Commercial kits of column tests for pre-transfusion testing have progressively replaced conventional tube tests in most laboratories. Aim of this study was to compare three commercial test cell panels for the identification of irregular red blood cell (RBC) alloantibodies. Overall, 44 samples with a positive indirect antiglobulin test (IAT) by routine testing were used for comparison of following panels: Ortho RESOLVE^®^ panelC (Ortho Clinical Diagnostics (OCD), Milan, Italy), ID-DiaPanel(-P) (Bio-Rad Laboratories, CA, USA) and Identisera Diana(P) (Grifols, Barcelona, Spain). Column agglutination techniques were used, with microtubes containing either microgel (Bio-Rad/Grifols) or glass bead microparticles (Ortho).

**Results:**

Alloantibody identification was possible in 38 samples, of which identical identification was shown in 33 samples by all methods. The remaining samples showed differences between certain methods, with the gel card system being superior to the glass card system for analyzing stored samples Considering that not all samples were evaluated in all three methods, the concordance rate reached 100% between Bio-Rad and Grifols, 90.5% between Bio-Rad and OCD, 86.5% between OCD and Grifols and 90.5% between all methods. Although differences in sensitivities were seen for specific antibodies, the three methods showed comparable performance for the identification of RBC alloantibodies.

## Introduction

The screening and identification of red blood cell (RBC) alloantibodies is performed as pre-transfusion testing (Type and Screen) and in pregnancy to detect potential hemolytic disease of the fetus and newborn (HDFN). A low ionic strength solution (LISS) indirect antiglobulin test (IAT) is considered the most suitable for the detection of clinically significant antibodies because of its speed, sensitivity and specificity [1, 2]. According to several international guidelines (such as BSH (British Society for Haematology) and CBO (‘Centraal Begeleidingsorgaan’) [3], the screening cell set must answer to certain requirements such as the inclusion of at least one cell with homozygous expression of the Fy^a^, Fy^b^ (Duffy antigens), Jk^a^, Jk^b^ (Kidd antigens), S and s antigens (MNSs antigens) and heterozygous expression for the K (Kell) antigen [4, 5]. If the antibody screen is negative, it can be predicted that more than 99% [2, 6] of the RBC units electronically matched for ABO groups will be compatible in the crossmatch (XM) test [7]. A positive antibody detection test is followed by the determination of the antibody specificity and the assessment of its clinical significance. An identification panel must contain RBC from group O donors with at least two phenotypes lacking and at least two phenotypes expressing the corresponding antigen (K, k, Jk^a^, Jk^b^, S, s, Fy^a^ and Fy^b^) [3, 5].

Several studies already addressed the comparison of commercial test cell panels for the detection of RBC alloantibodies. However, only few studies have focused on differences in identification of those RBC alloantibodies. Chang et al. [8], Roback et al. [9] and Taylor et al. [10] compared test cell panels form Bio-Rad and Grifols for RBC antibody identification. Garozzo et al. [11] compared results with test cell panels from OCD with those from Immucor. Sawierucha et al. [12] compared Bio-Rad with OCD. Cid et al. [13] compared, as in our study, the cell panels from Bio-Rad, Grifols and OCD. The aim of our study was the comparison of three test cell panels for the identification of irregular RBC alloantibodies; Ortho RESOLVE^®^ panel C from Ortho BioVue^®^ System, ID-DiaPanel and ID-DiaPanel P from Bio-Rad and Identisera Diana and Identisera Diana P from Grifols.

## Main text

### Materials and methods

#### Study design

The main objective was to determine the performance of three test cell panels in identifting clinically relevant antibodies. Concordance rates were calculated according to the CLSI (Clinical & Laboratory Standards Institute) guideline [14].

#### Samples

Samples (n = 44) for this study were collected from August 2016 until January 2018 and include ethylene-diamine-tetraacetic acid (EDTA) plasma or serum from pre-transfusion testing with a positive screening result (OCD: n = 33, Bio-Rad: n = 11). Screening testing was done with corresponding 11-cell identification panel from OCD or Bio-Rad, using untreated and papain-treated RBC. Within 5 days after specimen collection or after being stored in a frozen state by − 20 °C, the samples were also tested with the remaining methods (OCD/Bio-Rad and/or Grifols), if possible according to the available sample volume.

#### Reagents

Ortho BioVue System Poly Cassettes and Bio-Rad ID-Cards “LISS-Coombs” are comprised of six columns, while Grifols DG Gel Cards are eight column gel cards. Each microtube contains a wide reaction chamber in the upper part and an anti-human globulin (AHG) in the lower part. Cards from Ortho include a glass microbead matrix while cards from Bio-Rad and Grifols consist of a cross-linked gel (Sephadex) for the separation of agglutinated RBC. Additionally for the Ortho system, Ortho BLISS (a low ionic strength solution (LISS) designed to provide optimal ionic strength for antibody uptake) is to be added. All three systems also have ‘Neutral Cards’ which contain neutral gel suspension to perform saline and enzyme techniques (Ortho BioVue System Neutral Cassettes from OCD, NaCl enzyme test and cold agglutinins cards from Bio-Rad and DG Gel Neutral cards from Grifols). The following sets of reagent RBC were used: 3-5% resolve C (OCD), 0.8% ID-Diapanel 1-1 (BioRad) and 0.8% Identisera Diana (Grifols).

#### Principle of procedure

All three methods use the column agglutination technique. Agglutinated RBC are trapped in the gel or glass beads in the presence of irregular antibodies. According to the reaction pattern and the antigen configuration (displayed on an antigen table), the antibody present can be identified. In all samples, an autocontrol (AT; method testing the patient’s own red cells) must be included to make a difference between auto- and alloantibodies [15–18].

The Grifols analyses were performed automatically (Erythra^®^, Grifols) while the analyses by the other two methods were completed manually. Qualified laboratory technologists performed all tests in strict adherence to the manufacturer’s instructions. All reactions were read carefully with the aid of an illuminated box by at least two individuals.

#### Statistical analysis

All statistical analyses were conducted using the Microscoft Excel + Analyse-it^®^ software for Windows 10 (Analyse-it, Leeds, United Kingdom). Concordant results were measured and Cohen’s kappa coefficient (κ) was assessed to compare the ability to detect RBC alloantibodies by the three methods.

### Results

Out of 44 samples, 21 were investigated with all three methods. 23 samples were investigated with only two methods because of insufficient sample volume; seven samples with Bio-Rad and Grifols, and 16 samples with OCD and Grifols reagents (Additional file 1: Figure S1). In 38 samples at least one alloantibody was identified. An identical identification was found in 33 out of 38 samples. In four samples additional alloantibodies were found by a certain method; in two samples (tested with all three methods) an additional anti-D (Rhesus antigen), anti-Lu^a^ (Lutheran antigen) and anti-C (Rhesus antigen) were discovered by Bio-Rad and Grifols (Table 1; sample C, D) and in two samples (tested with only OCD and Grifols) an additional anti-D was found by Grifols (Table 1; sample F, G). In those last two samples however, enzyme treated cells were necessary for the detection of the additional antibodies. In one of the remaining samples, anti-Kp^a^ was not identified by OCD as Kp^a^ was not present in the test system (Table 1; sample A). Results of two other samples were considered inconclusive because of insufficient sample volume (Table 1; sample B, E). An overview of these results is shown in Table 1.

**Table 1:**
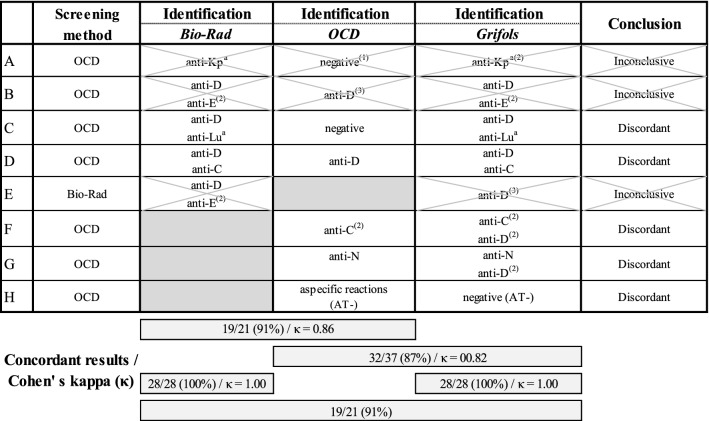
Overview of discordant/inconclusive results

^(1)^The antigen was not present in this test system

^(2)^Reaction only seen in enzyme-treated cells

^(3)^No possibility of using enzyme-treated cells because of insufficient sample volume

Overall, the use of the described panels enabled the identification of 54 antibodies. The most frequently identified antibodies were anti-D (24.1%) and anti-E (16.7%), followed by anti-C (9.3%) and anti-c (9.3%). Table 2 reports the antibody specificities.

**Table 2:** Overview of the antibody specificities

Specificity	n (%)	n (Bio-Rad)	n (OCD)	n (Grifols)
anti-D	13 (24.1)	8	7	13
anti-E	9 (16.7)	7	7^(1)^	8^(2)^
anti-C	5 (9.3)	3	3	5
anti-c	5 (9.3)	4	4	5
anti-K	4 (7.4)	2	3	4
anti-Fy^a^	4 (7.4)	1	4	4
anti-M	3 (5.6)	2	2	3
anti-Le^a^	3 (5.6)	2	3	3
anti-Jk^a^	2 (3.7)	1	2	2
anti-C^w^	1 (1.9)	1	1	1
anti-Kp^a^	1 (1.9)	1	n.t.^(3)^	1
anti-Lu^a^	1 (1.9)	1	0	1
anti-N	1 (1.9)	0	1	1
anti-S	1 (1.9)	0	1	1
anti-s	1 (1.9)	1	1	1
Total	54 (100)	34	39	53

^(1)^Anti-E was not detected in one sample with OCD because of insufficient sample volume

^(2)^Anti-E was not detected in one sample with Grifols because of insufficient sample material

^(3)^The antigen Kp^a^ was not present in this test system

Six samples showed unexplained reactions in all methods used; nonspecific agglutination (reaction in only a few cells of the panels used), pan agglutination (positivity in all the cells of the panels used) or no reaction in any cell. One sample showed unexplained reactions with one method and a negative result with the other method and was therefore considered discordant (Table 1; sample H). In these six samples, three samples showed a positive AT result and three samples a negative result.

In addition to finding the antibody specificity in the samples, the presence of underlying antibodies with possible clinical significance must be excluded. Applying the rules defined by the BSH guidelines [3], underlying antibodies could not be excluded in 10 out of 28 samples tested with Bio-Rad (35.7%), in 13 out of 37 samples tested with OCD (35.1%) and in 14 out of 44 samples tested with Grifols (31.8%). Those antibodies mostly belonged to blood group systems with significant clinical importance (Rhesus-, Kell-, Duffy-, Kidd- and MNS-blood group systems).

### Discussion

As in Chang et al. [8], Cid et al. [13] and Garozzo et al. [11], the most frequently identified antibodies were anti-D (24.1%), in direct relationship with antenatal prophylaxis against haemolytic disease of the newborn, and anti-E (16.7%). Anti-K represented only 7.4% in our study while a greater percentage was described in Roback et al. [9] and Tayler et al. [10]. The concordance rate between Bio-Rad and Grifols is 100%, between Bio-Rad and OCD 91%, between OCD and Grifols 87% and between all three methods 91%. These are similar percentages compared to the described articles above (Table 3).

**Table 3:** Overview literature

	Test cell panels	IAGT identification no. of samples/concordant results	Concordance rate (%)
Taylor et al. [10]	Bio-Rad vs Grifols	361/328	90.86
Cid et al. [13]	Bio-Rad vs Grifols	26/25	96.15
	OCD vs Grifols	26/24	92.31
	Bio-Rad vs OCD	26/23	88.46
	Bio-Rad vs OCD vs Grifols	26/23	88.46
Garozzo et al. [11]	OCD vs Immucor	78/74	94.87
Chang et al. [8]	Bio-Rad vs Grifols	51/50	98.04
Roback et al. [9]	Bio-Rad vs Grifols	759/759	98.68
Sawierucha et al. [12]	Bio-Rad vs OCD	165/226	73.00
Blomme et al. (2019)	Bio-Rad vs Grifols	28/28	100.00
	OCD vs Grifols	32/37	86.49
	Bio-Rad vs OCD	19/21	90.48
	Bio-Rad vs OCD vs Grifols	19/21	90.48

Bio-Rad identified three additional antibodies in comparison with OCD (an anti-D, anti-Lua and anti-C), and Grifols identified five additional antibodies in comparison with OCD (an anti- Lua, anti-C, and three times anti-D). It should be noted that anti-C can be missed when anti-D is present simultaneously and when the heterozygous cell for the antigen C (i.e. Cc) is not positive. This is because the homozygous cells for the antigen C (i.e. CC) overlap those with antigen D. The Bio-Rad, OCD and Grifols identification panels showed unexplained reactions in six samples. In three of those samples the AT was positive, suggesting the presence of autoantibodies [3, 19]. In the other three samples, the presence of a private antigen was not excluded. As far as the specificity of the antibodies, no equal identification rate was obtained with the three methods: the most antibodies were detected with Grifols and Bio-Rad, although sometimes the use of enzyme treated cells was necessary for the identification of the additionally discovered antibodies. The glass card method seems to be comparable to the gel card method in RBC antibody screening, although in stored samples, antibodies were more frequently detected with the gel card system.

The exclusion of underlying antibodies was not possible in a similar percentage of samples with all three methods. So it is important to recognize the limitations of the panel in use. A single panel may not permit identification of some common combinations of antibodies. A selection of two different panels increases the probability of being able to identify a mixture of antibodies. Additional techniques, for example the use of a panel of enzyme treated cells, can also be helpful in antibody identification and is strongly recommended for antibody identification, particularly when an antibody is weakly reactive with the antiglobulin technique, or when a mixture of antibodies is present [3]. We examined if there was a method that gave an advantage in the identification of the antibody as to the absence of additional nonspecific reactions making identification more difficult, the unnecessity of using additional techniques like enzyme treated panels and the positivity of both homozygous and heterozygous cells. Each method has a comparable degree of ‘difficulties’ concluding that there is no manifest advantage for one particular method in case of facilitation of antibody identification.

### Conclusion

Three test cell panels for identification of irregular RBC antibodies were compared: Autovue^®^ Innova (Ortho Clinical Diagnostics (OCD), Milan, Italy), ID-GelStation (Bio-Rad Laboratories, CA, USA) and Erytra^®^ (Grifols, Barcelona, Spain). The resulting antibody identifications showed subtle differences between the three methods, with the gel card system (Bio-Rad and Grifols) being superior to the glass card system (Ortho) for analyzing stored samples. However, no manifest advantage for a particular method in case of facilitation of antibody identification was found. In conclusion, all three systems were considered reliable and safe for routine testing in the immunohematology laboratory.

## Limitations

Only one manufacturing lot number of a test cell panel was tested for each firm, which can also explain certain differences. In our study, there is also no adjustment for the fact that the expression of RBC antigens on each reagent cell in a panel from one manufacturer is not the same as the expression on the RBC’s in a panel from another manufacturer.

## Supplementary information

**Additional file 1: Figure S1.** Overview tested samples.

## Data Availability

The datasets used and/or analyzed during the current study are available from the corresponding author on reasonable request.

## Abbreviations

AT: Autocontrol
BSH: British Society of Haematology
CBO: ‘Centraal Begeleidingsorgaan’ (The Netherlands)
CLSI: Clinical & Laboratory Standards Institute
D, C, c, E, C^w^: Rhesus antigens
EDTA: Ethylenediaminetetraacetic acid
Fy^a^, Fy^b^: Duffy antigens
HDFN: Hemolytic disease of the fetus and newborn
IAT: Indirect antiglobulin test
Jk^a^, Jk^b^: Kidd antigens
K, k, Kp^a^: Kell antigens
Κ: Cohens’s kappa coefficient
Le^a^: Lewis antigen
LISS: Low ionic strength solution
Lu^a^: Lutheran antigen
OCD: Ortho Clinical Diagnostics
RBC: Red blood cell
