# Supplementary figures and images for: A comparison of three column agglutination tests for red blood cell alloantibody identification
Source: BMC Res Notes. 2020 Mar 4;13:129. doi: 10.1186/s13104-020-04974-x (PMC7057655; doi:10.1186/s13104-020-04974-x)

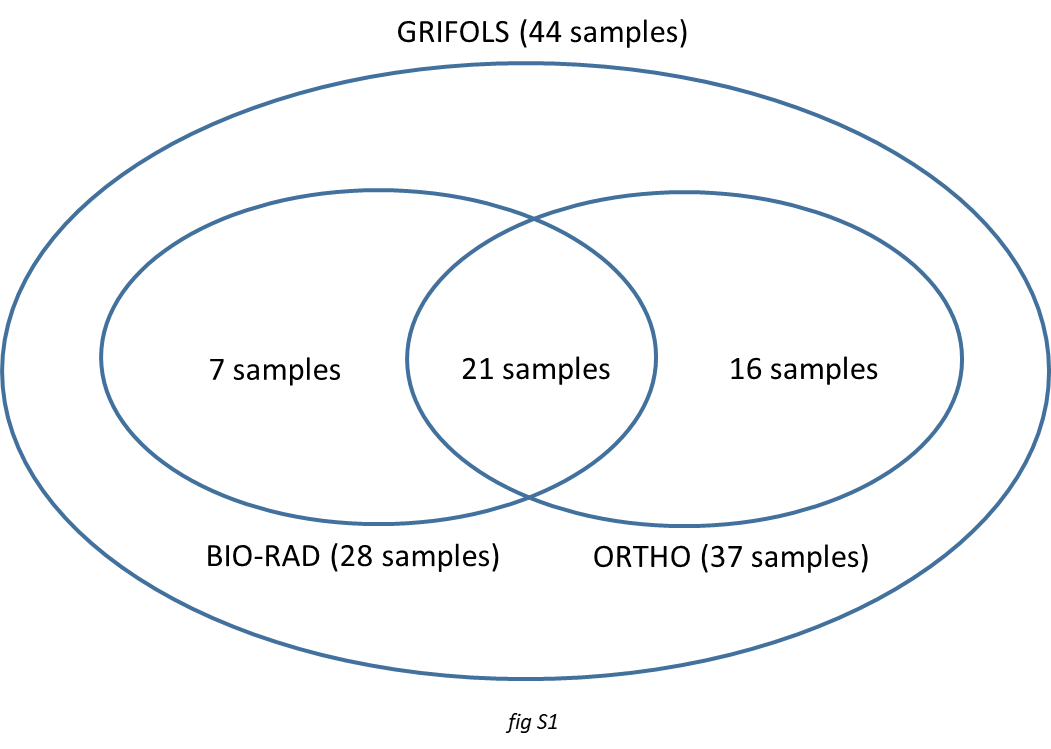

Supplement: Supplementary file 1 — Additional file 1: Figure S1. Overview tested samples. [file 13104_2020_4974_MOESM1_ESM.docx]
